# Optimizing Cardiac Inpatient Flow and Resource Allocation in Low‐Resource Settings Using Queuing Theory: Insights From a Tertiary Hospital in Bangladesh

**DOI:** 10.1002/puh2.70304

**Published:** 2026-06-19

**Authors:** Md. Borhan Uddin, Khalidur Rahman, Md. Asaduzzaman, Sabbir Tahmidur Rahman, Md. Sabbir Hossain, Md. Shihab Mostafa

**Affiliations:** ^1^ Department of Statistics Shahjalal University of Science and Technology Sylhet Bangladesh; ^2^ Department of Medicine Sylhet MAG Osmani Medical College Hospital Sylhet Bangladesh

**Keywords:** cardiac care, hospital management, inpatient flow optimization, low‐resource settings, queuing theory, resource allocation

## Abstract

Healthcare systems in low‐resource settings face persistent challenges in providing timely cardiac care. This study applies queuing theory models (*M*/*M*/*c*/*c* and *M*/*M*/*c*/*K*) to optimize inpatient flow and resource allocation in the cardiology department of Sylhet MAG Osmani Medical College Hospital, Bangladesh. Using data from 6277 patients treated in 2023, the study analyzes arrival patterns, lengths of stay, and bed capacities across four units: Normal Care Unit (NC), Cath Lab (CL), Coronary Care Unit (CCU), and Progressive Coronary Care Unit (PCCU). The results reveal critical shortages in resources and operational bottlenecks, particularly in the CCU and PCCU, where bed shortages frequently compromise care quality. Recommendations include reallocating underutilized resources, such as beds from the CL, and expanding capacities in the CCU from 8 to 15 beds and in the PCCU from 10 to 25 beds to accommodate unmet demand. The study also highlights the detrimental effects of overcrowding and the unregulated use of extra beds in the NCU, proposing strategies to enhance operational efficiency and patient accessibility. This research provides data‐driven strategies to reduce congestion, enhance patient outcomes, and promote healthcare equity in low‐resource settings, addressing the unique challenges of underserved populations in developing countries while contributing to the broader goal of strengthening healthcare systems and advancing global health equity.

## Graphical abstract

This study applies queuing theory models (M/M/c/c and M/M/c/K) to optimize inpatient flow and resource allocation in the cardiology department of Sylhet MAG Osmani Medical College Hospital, Bangladesh. Using data from 6,277 patients in 2023, it identifies bottlenecks in CCU and PCCU, highlights bed shortages and overcrowding, and proposes reallocating resources and expanding capacities to improve efficiency, patient outcomes, and healthcare equity in a low‐resource setting.

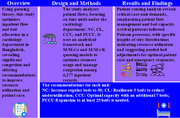

## 1 Introduction

Cardiovascular diseases, especially ischemic heart disease, are among the leading causes of death worldwide and place a significant burden on healthcare systems globally [1, 2]. Although developed nations have significantly reduced heart attack mortality rates, countries like Bangladesh continue to face increasing challenges due to limited resources and systemic inefficiencies [3, 4]. In such settings, access to adequate cardiac care is often hindered by insufficient facilities, overcrowding, poor management practices, and underutilized resources [5, 6].

Several simulation and optimization techniques have been used to address hospital congestion and enhance operational efficiency. For instance, discrete‐event simulation combined with design of experiments (DES–DOE) frameworks has been applied to optimize turnaround times and patient flow in internal medicine clinics, whereas goal‐driven admission control models have been developed to alleviate emergency department congestion and streamline inpatient transfer processes [7, 8]. However, these approaches are often limited to single‐unit systems or rely heavily on data and computational resources, making them less practical in complex, multiunit hospital environments, particularly in resource‐constrained settings.

Queuing theory, a branch of operations research, offers a simpler but analytically robust alternative for evaluating and reducing congestion in systems such as transportation networks, crowd management, and telecommunications [9, 10, 11, 12]. In healthcare, it has been widely implemented in developed countries to improve facility management, enhance resource allocation, and reduce bottlenecks, including those related to hospital bed management [13, 14, 15, 16, 17]. However, its application in low‐resource healthcare settings in developing countries remains relatively underexplored.

Key factors, such as patient arrival rates and lengths of stay, are critical for determining service capacity and overall system performance in healthcare systems [18, 19]. Developing countries face unique operational constraints and patient flow patterns that differ from those in high‐income settings, making it essential to adapt analytical approaches accordingly.

The healthcare system in Bangladesh presents distinct structural challenges, including severe bed shortages, informal overflow practices (e.g., floor beds), weak referral systems, and heavy reliance on public tertiary hospitals. These factors substantially influence patient flow dynamics. To our knowledge, this study is the first to apply the *M*/*M*/*c*/*K* and *M*/*M*/*c*/*c* queuing models to assess the impact of additional beds and overflow practices on healthcare system performance in Bangladesh. The findings provide empirical insights into patient flow dynamics and offer actionable recommendations—such as resource redistribution and targeted capacity expansion to enhance access, efficiency, and equity in care.

## 2 Methods

### 2.1 Study Setting

The study was conducted in the cardiology department of Sylhet MAG Osmani Medical College Hospital [20], a prominent tertiary healthcare institution serving a large, predominantly low‐income population in the north‐eastern part of Bangladesh. The hospital, located in the secondary city of Sylhet, caters to over 9 million people from the Sylhet division, providing 24‐h services across 18 departments, including the cardiology department, which is crucial for managing cardiovascular diseases.

Inpatients are admitted through the hospital's admission department, which directs them to the appropriate departments. Admitted patients may be rerouted within the departments, if necessary. Within the cardiology department, patients are managed through four distinct units, each designed to handle specific types of inpatient flows: Normal Care Unit (NC), Cath Lab (CL), Coronary Care Unit (CCU), and Progressive Coronary Care Unit (PCCU).

- **NC unit**: The NC unit handles primary flow patients, accounting for over three‐fourths of the total inpatient flow in the cardiology department. Patients admitted here typically have non‐acute cardiac conditions such as chronic stable angina, stable heart failure, or valvular heart disease. Most patients are discharged directly from the NC unit, but a small percentage may be transferred to the CCU for intensive care if their condition worsens.
- **CL**: This unit is also equipped to provide a check to a particular group of patients from the primary flow. Here, the cardiac patients are admitted for coronary angiograms. All patients generally leave this unit with a short length of stay (LOS) and are transferred to the PCCU before final discharge. In rare cases, patients may be transferred to the CCU if they require more intensive care.
- **CCU**: The CCU is designated for critical patients, primarily those in the secondary flow with severe acute heart conditions like acute myocardial infarction, arrhythmia, or acute left ventricular failure. After receiving intensive care, patients are usually moved to the PCCU, though some may be referred to more specialized hospitals in the capital city of Dhaka for advanced treatment.
- **PCCU**: The PCCU is mainly for patients transitioning from the CCU after their treatment there. Once the subsequent intense observation is done, the patient is allowed to leave the hospital. In occasional cases, some patients are transferred back to the CCU when their conditions become worse. Patients with crescendo angina or like diseases are directly admitted to the PCCU. Besides, a significant portion of emergency patients are also often admitted to the PCCU because of the unavailability of beds at CCU. As mentioned earlier, almost all patients in the CL unit are routed to this unit before final discharge.

**FIGURE 1:**
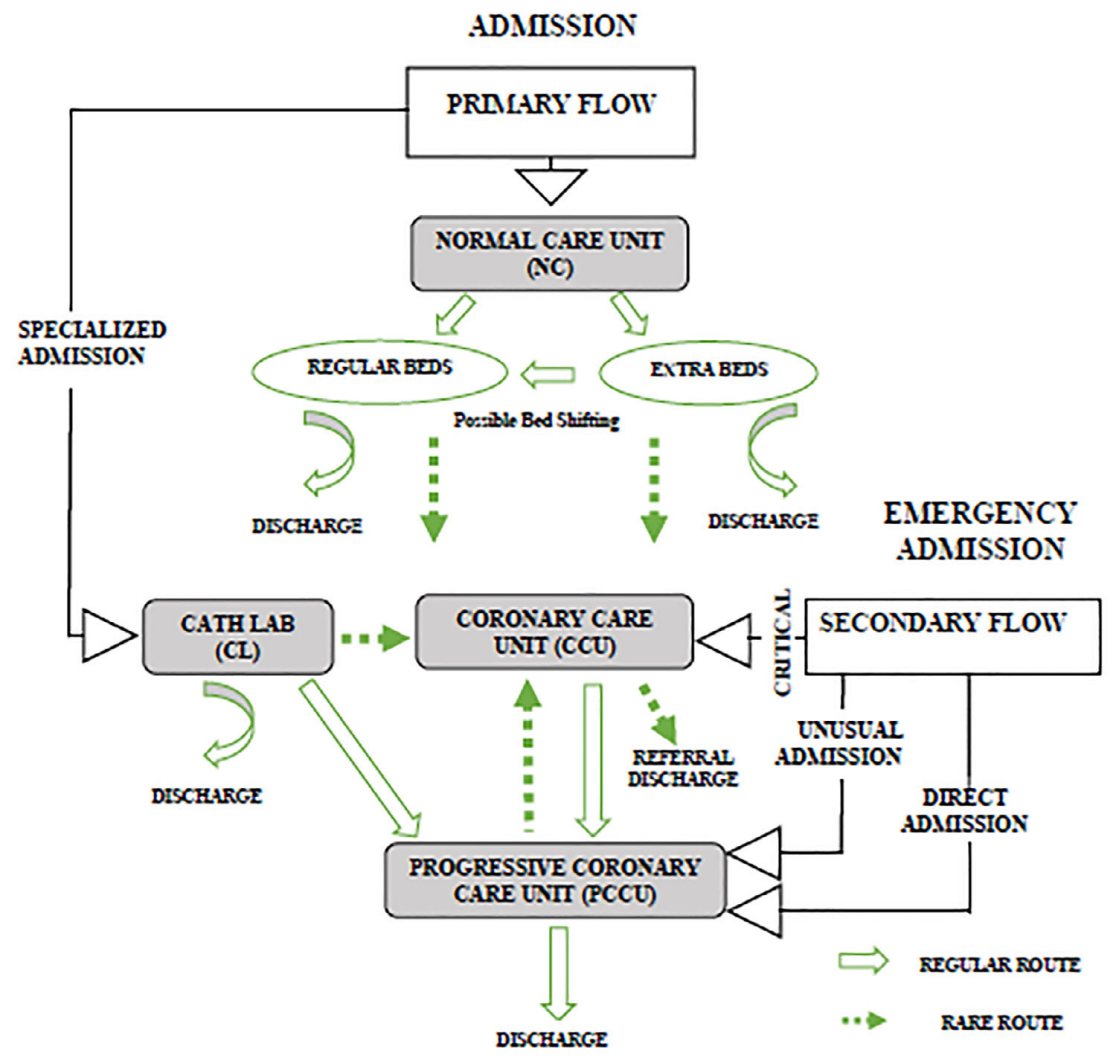
Flow chart of inpatient flows.

Figure 1 portrays the routing of two major patient flows at the cardiology department. The real scenario is too complex to visualize the configuration of all possible patient flows in a simple form. Several medical and nonmedical factors are simultaneously operative, so it is impossible to recognize the complete routing network. For simplicity, while retaining the principal integrity, it is assumed that the nonobligatory flows have a negligible impact on the overall management of inpatient care. The current study aims to uncover resource allocation inefficiencies within the abovementioned healthcare units, proposing queuing theory‐based solutions to address them.

### 2.2 Data Collection and Cleaning

The inpatient data for the cardiology department were stored electronically by a designated operator. This study examines the inpatient flows within this department during 2023, with the necessary permissions obtained for data collection. In the assessment year, the cardiology department treated 6411 patients. However, data pertaining to hospital staff receiving special treatment and patients in private cabins were excluded, resulting in a final dataset of 6277 patients. Additionally, records for 291 patients, which lacked admission time, were excluded from the time‐varying arrival pattern analysis. Approval and necessary permissions were obtained for the use of anonymized patient data.

### 2.3 Analytical Framework for Optimizing Resource Usage and Managing Congestion

This study employed queuing theory models, specifically *M*/*M*/*c*/*c* and *M*/*M*/*c*/*K*, to analyze inpatient flow dynamics and optimize resource utilization within the cardiology department. The *M*/*M*/*c*/*K* model represents a system in which patient arrivals follow a Poisson process (Markovian), service times are exponentially distributed, there are *c* parallel servers (beds), and the total system capacity is limited to *K* patients (including both those receiving service and those waiting). The *M*/*M*/*c*/*c* model represents a loss system with no waiting space, where arriving patients are blocked if all servers (beds) are occupied. The focus was on improving resource allocation and managing congestion in inpatient flows. Congestion in hospital units arises and resolves under distinct conditions. The forms of congestion under normal and emergency conditions are not the same.

Although a hospital or healthcare unit performance is shaped by multiple operational and organizational factors such as staffing levels, discharge policies, and admission prioritization, this study focuses on three core determinants—patient arrival pattern, LOS, and the number of available beds—because these factors form the fundamental structural components of any queuing system and directly determine demand, service rate, and system capacity. Similar simplifications have been adopted in prior hospital queuing studies and DES–DOE frameworks, where system‐wide performance is primarily driven by similar factors [7, 14, 17].

The exclusion of additional factors does not imply irrelevance; rather, it reflects a modeling trade‐off to maintain analytical tractability and ensure parameter identifiability using available hospital data. Factors such as staffing and admission policy are implicitly reflected in LOS and effective service rates, whereas discharge policies are partially embedded in observed throughput.

The patient flow that arrives in a healthcare unit represents the demand. In the same context, the capacity of the unit is linked with the LOS and the available beds in it. Addressing the demand for arrival and the capacity of the unit (supply) determines the operational characteristics and the performances of a healthcare unit under investigation. It is always promising if any performance measure of a healthcare unit could be expressed based on the aforesaid three factors. In that case, when two of the three factors are recognized, the management can determine the third one to meet the performance target. Again, by varying the target level of performance for the specified values of any two factors, the value of the third factor is easily determined.

Understanding arrival patterns is critical to analyzing how patient inflow affects healthcare unit dynamics. This analysis helps identify the effective arrival rate, known as the throughput, to prevent unnecessary patient overflow, which is impossible to provide healthcare facilities within in a low‐resource setting and exceeds the facility's capacity. It also informs decisions about the need for additional healthcare infrastructure. Similarly, determining the optimal number of beds supports cost‐effective facility sizing and design of healthcare facilities.

To evaluate performance and optimize configurations, metrics, such as steady‐state probabilities, throughput, and blocking probabilities, are useful. These measures facilitate the identification of optimal bed allocations and support resource planning for enhanced operational efficiency.

#### 2.3.1 Analytical Queuing Model Selection

Queuing theory was chosen over purely simulation‐based or system dynamics approaches because it provides closed‐form expressions for key performance measures such as blocking probability, utilization, and throughput, which are essential for capacity planning. Although DES and hybrid models can capture additional system complexity, they often require extensive parameter calibration and do not easily yield interpretable analytical thresholds for bed optimization. In contrast, queuing models enable a direct evaluation of system performance under varying capacity configurations, making them particularly suitable for low‐resource settings where rapid and interpretable decision support is required.

In this study, to select the analytical queuing models for understanding the stochastic nature of patient flows, we can describe a healthcare unit as a station where patients are served. We can also consider the available beds in the unit as servers, providing services to the patients by facilitating their stay. A patient can be both an output and an input to the queuing system within the unit.

**FIGURE 2:**
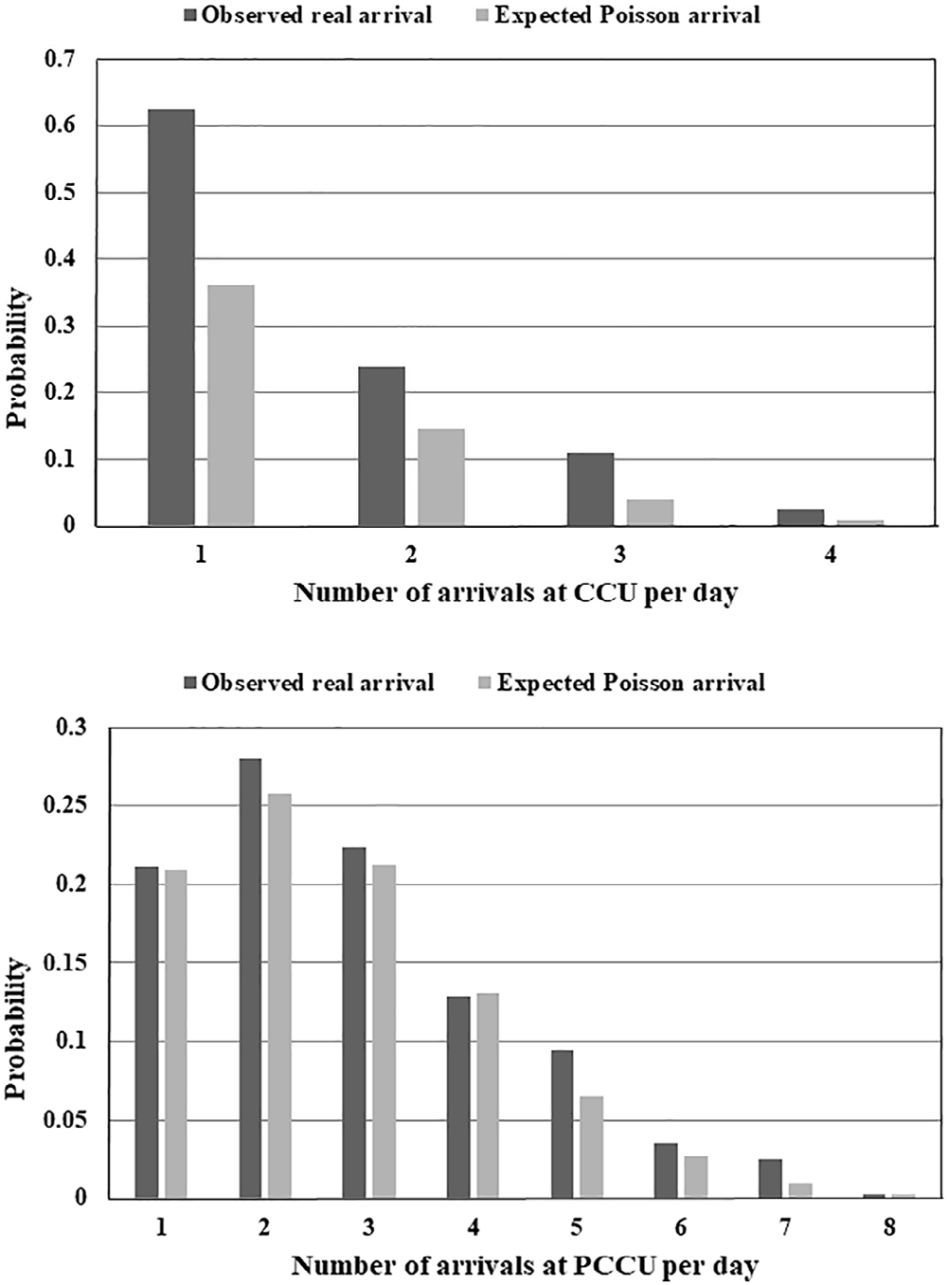
Distributions of per‐day patient arrivals at CCU and PCCU. CCU, Coronary Care Unit; PCCU, Progressive Coronary Care Unit.

We can assume that the patients get admitted to each unit according to the Poisson process with a certain rate, λ, and the inter‐arrival time, *M*, follows the exponential distribution. The assumption of a Poisson arrival process is justified based on both empirical observation and established healthcare operations literature. In hospital systems, patient arrivals are typically independent events driven by random external health incidents, which align well with Poisson assumptions. Prior studies have demonstrated that emergency and inpatient arrivals often follow Poisson distributions [14, 17], particularly when aggregated over daily or hourly intervals. In this study, as discussed in Section 3.2 and indicated in Figure 2, the observed near‐equality of mean and variance in arrival data further supports this assumption.

Note that average length of stay (ALOS), μ, is a key measure of the quality of service (service rate) provided to the patients in a unit and, as such, determines the effectiveness of the unit. Besides, because the LOS of a patient is mainly influenced by their personal attributes (e.g., severity of disease, age, place of residence, etc.), the inclusion of common attributes in the modeling can be done by bringing the LOS to the corresponding congestion models. As noted in Section 3.3, the distributional form of the LOS of each unit is not unique; rather, it is a general distribution *G*. If there are *n* beds (servers) in the unit, we will have the overall service rate for *n* patients as

(1) $$\mu_{n}=\frac{n}{\mu }$$

This service rate is very essential to determining the steady‐state probabilities of the different numbers of patients under the treatment facility of a healthcare unit. Obviously, the relevant queuing models are state‐dependent as an adjustment in the number of beds in the unit as well as the patient's personal attributes will change the service rate of the healthcare facility.

#### 2.3.2 Unit‐Specific Model Applications

The *M*/*M*/*c*/*c* model should be applied to healthcare units without buffer capacity, whereas the *M*/*M*/*c*/*K* model should be used for units accommodating overflow patients. These models are valid to provide insights into the probability of service denial (blocking), system throughput, and the optimal number of beds required to meet patient demand.

##### 2.3.2.1 Cath Lab

Patients that arrive at the CL unit when its beds (*c*) are fully occupied are generally unable to get admission to it. That is, there is no opportunity to build the patient queue with additional beds, and it operates without any additional buffer beds. Therefore, the total number of patients that are allowed in the entire queuing system of this unit is equal to the number of beds in it. Thus, in Kendall's notation [21], the queuing model under this inquiry would be *M*/*M*/*c*/*c* or *M*/*G*/*c*/*c*. Notice that *M* denotes a Markovian (Poisson/exponential) process, whereas *G* denotes a general distribution. It is to be noted that *M*/*M*/*c*/*c* and *M*/*G*/*c*/*c* state‐dependent queues are stochastically identical [22], and thus the *M*/*M*/*c*/*c* model may be used to find the limiting probabilities for the number of patients in the system. Thus, we can get the steady‐state probabilities that *n* beds are occupied as

(2) $$p_{n}=\frac{\frac{{\left(\lambda \mu \right)}^{n}}{n!}}{\sum_{i=0}^{c}\frac{{\left(\lambda \mu \right)}^{i}}{i}},\;n=0,1,2,\;\text{…},c$$

and the probability that an arriving patient is unable to admit the unit (blocking occurs) is given by

(3) $$p_{\text{Balk}}=\frac{\frac{{\left(\lambda \mu \right)}^{c}}{c!}}{\sum_{i=0}^{c}\frac{{\left(\lambda \mu \right)}^{i}}{i!}}$$

Thus, the throughput of the patient flow at the unit is

(4) $$\Theta =(1-p_{\mathrm{Balk}})\lambda$$

In terms of hospital facilities, throughput is defined as the rate of patient flow that has no reason to refuse admission to any patient [14].

##### 2.3.2.2 NC Unit

The highest number of patients arrives at NC. As noted in Section 3.1, in addition to the regular beds (*c*), a number of extra beds (*K*) are provided to meet the requirements of the patients. In the terminology of queuing theory, these extra bed facilities could be perceived as the buffering space in the healthcare system of the unit. Thus, in the light of the arguments provided in Section 2.3.2.1, for Poisson‐based arrival and general distribution of LOS, the patient flow of this largest unit can be modeled as an *M*/*M*/*c*/*K* queuing model. Here, the additional assumption is that after the full occupation of regular beds, the overall service rate, as can be estimated using Equation (1), remains unchanged. This assumption is logical in the sense that no additional staff is usually provided to serve the extra patients. Accordingly, the corresponding probabilities of different numbers of patients in the unit are as follows [23]:

(5) $$p_{n}=\left\{\begin{array}{c} \frac{{\left(\lambda \mu \right)}^{n}}{n!}p_{0}\;\;\;\;\;\;\;\;\;\;\;\;\;\;\;\;\;\;\;\;\;\;\;\;\;\;\;\;\;\;\;\;\text{for}\;\;1\leq n\leq c\\ \frac{{\left(\lambda \mu \right)}^{n}}{{\left(c\right)}^{n-c}c!}p_{0}\;\;\;\;\;\;\;\;\;\;\;\;\;\;\;\;\text{for}\;\;c\leq n\leq K \end{array}\right.$$

and

(6) $$p_{0}={\left(1+\sum_{i=1}^{c-1}\frac{{\left(\lambda \mu \right)}^{i}}{i!}+\sum_{i=c}^{K}\frac{{\left(\lambda \mu \right)}^{i}}{{\left(c\right)}^{i-c}c!}\right)}^{-1}$$

Clearly, the balking probability at the highest capacity, *K*, is

(7) $$p_{\text{Balk}}=\frac{\frac{{\left(\lambda \mu \right)}^{K}}{{\left(c\right)}^{K-c}c!}}{\left(1+\sum_{i=1}^{c-1}\frac{{\left(\lambda \mu \right)}^{i}}{i!}+\sum_{i=c}^{K}\frac{{\left(\lambda \mu \right)}^{i}}{{\left(c\right)}^{i-c}c!}\right)}$$

and the throughput at the unit can correspondingly be calculated using Equation (4).

##### 2.3.2.3 CCU and PCCU

The number of beds (*c*) at CCU and PCCU is fixed. When all beds are occupied, there is no way to allow additional patients to be admitted. Therefore, like the CL unit, the *M*/*M*/*c*/*c* queuing model is appropriate to be used to model the patient flow at these units. So Equations (2)–(4) can be utilized to calculate the probabilities of patients and the throughputs, respectively. Note that if the real arrival rate of CCU is $\lambda_{1}$, then the refused portion of patients, $\lambda_{1}p_{\mathrm{Balk}}$, are unusually routed to PCCU.

As the main goal of this study is to help in delivering and promoting the intervention to prevent heart attacks, we need to develop a CCU unit where the abovementioned refused portion of emergency patients will not be declined or redirected. By merging the unrestricted throughput from CL and accommodating all the patients treated at CCU as well as those directly admitted to PCCU, we also need to optimize the number of beds at CL and PCCU. In this case, conceptualizing a merging topological network of patient flows may be useful [24]. Besides, it is necessary to do search optimization at NC.

### 2.4 Model Validation and Verification

The proposed queuing models were validated through a combination of empirical consistency checks and expert evaluation. Parameter estimates, including arrival rates and length‐of‐stay distributions, were cross‐validated with hospital administrative staff to ensure operational realism. In addition, sensitivity analyses were conducted to assess model stability under varying bed capacities, confirming that the results remained consistent with expected logical behavior.

## 3 Results and Discussion

### 3.1 Patient Routing

Patient routing details have been outlined in Section 2.1 and visually represented by a flow chart in Figure 1. Table 1 quantifies the patient flows and bed capacities across the four units. On the basis of medical assessments, patients are directed to one of the units: NC, CL, CCU, or PCCU. The majority of patients require standard care at NC, whereas a smaller portion necessitates emergency care at CCU.

**TABLE 1:** Arrangement of beds and flows at cardiology department.

Unit	NC	CL	CCU	PCCU
No. of admission	4734	346	295	902
Percentage	75.42	5.51	4.7	14.37
No. of beds	Regular (including 4 paying)—24 Extra (observed maximum)—59	10	8	10

Abbreviations: CCU, Coronary Care Unit; CL, Cath Lab; NC, Normal Care; PCCU, Progressive Coronary Care Unit.

The importance of patient flow management in critical healthcare settings, emphasizing the need for adequate bed capacity and resource allocation to reduce congestion and improve patient outcomes, has been highlighted [25]. Another study [26] underscores the use of queuing theory in optimizing hospital resource management, which aligns with the approach used in this study in analyzing patient routing and bed allocation.

Note that, as a tertiary hospital, Sylhet MAG Osmani Medical College Hospital cannot refuse admission. Consequently, emergency patients often overflow into the PCCU due to chronic bed shortages at the CCU. This resource mismatch underscores systemic inefficiencies in meeting the demand for emergency cardiac care, which disproportionately affects low‐income patients who rely on public hospitals for affordable treatment. Such mismatch is also unexpected owing to the fact that the sooner a heart attack patient is accommodated and treated at the CCU, the better the chance is for the patient to recover and survive. This crisis epoch is termed the “Golden Hour” [27]. For this reason, the corresponding authorities and policymakers are required to place more emphasis on this emergency chain.

Moreover, unlike the standard practices in developed countries, a unique and challenging condition exists at NC, where additional patients are accommodated by placing extra beds on the floor within the healthcare unit. This practice leads to overcrowding and compromises the quality of care. Throughout the year, bed occupancy in each unit consistently hovers around 100%, highlighting an urgent need for effective resource reallocation and capacity expansion.

### 3.2 Arrival Patterns

On the basis of day‐wise data for 365 days in the year, the mean and variance of arrivals for CCU are, respectively, 0.81 and 0.92, whereas these values are 2.47 and 2.99 for PCCU. As the values of mean and variance for each unit are almost equal, these arrival patterns of secondary flow could be more precisely modeled by the Poisson process. The fitting of Poisson arrivals is shown in Figure 2. Earlier research demonstrated [28, 29] that patient arrival patterns in emergency departments and admit to hospital often follow the Poisson processes, supporting the validity of the models intended to in the current study.

It is also found that the per‐day number of arrivals of primary flow at NC and CL have mean values of 12.97 and 0.95, respectively. With the help of the data entry operator's assessment, it is estimated that around 20% and 10% of arrivals at PCCU are, respectively, rightly assigned to this unit and redirected from CCU patient flow. Consequently, the absolute arrival rate at PCCU should be considered at 0.49 patients per day. Similarly, the redirected flow at CCU is 0.25 patients per day. All these indicate that the demanded flow at CCU should be considered 1.06 patients per day.

**FIGURE 3:**
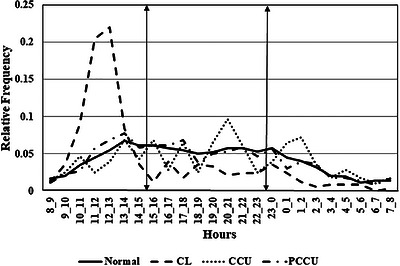
Variation in patient arrivals over a 24‐h period in each unit. CCU, Coronary Care Unit; CL, Cath Lab; PCCU, Progressive Coronary Care Unit.

Each unit has time‐varying arrivals during the 24 h of a day. In view of that, the arrival patterns of each unit over a 24‐h period have been demonstrated in Figure 3. According to the duty roster for medical staff and shifting hours, the whole day is divided into three intervals: 8:00–15:00, 15:00–22:00, and 22:00–8:00. It is obvious that the maximum workload regarding the arrival of patients is generated during the first shift (8:00–15:00), and thus supporting staff are allocated accordingly. The arrival rate is lowest overnight (22:00–8:00), caused by the unavailability of transportation at later times at night and in the early morning. However, we have been informed that the fluctuation of the arrival rate over 24 h does not considerably hinder the capacity of the cardiology department in terms of bed allocation. Hence, any decision‐making based on the average arrival rate per day is realistic to provide services to the inpatient flows.

Besides, the maximum arrival at CCU is observed between 19:00 and 21:00. By examining the records of the admitted patients during this time interval, it is evident that the incidence of heart attacks is more likely to occur in the morning [30], and the remote patients are late to be transferred to the tertiary level via primary and secondary levels of healthcare. Such delays reflect broader systemic inequities, where transportation and referral inefficiencies compound the risk for underserved populations. Addressing these disparities requires tailored operational strategies to reduce delays and allocate resources equitably.

Notice that, because a cardiology emergency department operates as a continuous 24/7 critical care service with comparable case urgency and consistent operational readiness throughout the week, variations between weekdays and weekends are not considered meaningful for analysis and have therefore been excluded from this study.

### 3.3 LOS Distributions

**FIGURE 4:**
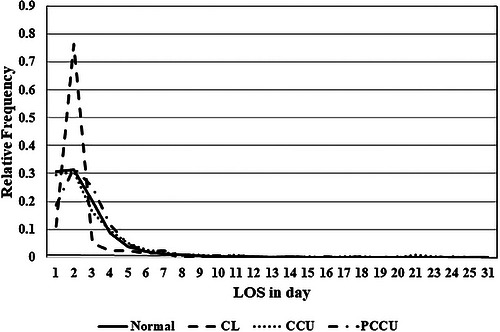
Distribution of LOS at each unit. CCU, Coronary Care Unit; CL, Cath Lab; LOS, length of stay; PCCU, Progressive Coronary Care Unit.

After getting admission to a unit, patients need to stay there to receive the treatments. The duration is termed the LOS and has been calculated by deducting the date of admission from the date of discharge. Its value depends on several factors, including the condition and need of a patient for receiving treatment and the administrative capacity to meet the requirements of the patient. The associated LOS data for all units are summarized in Table 2, and the distributions are depicted in Figure 4. The median of LOS at each unit is 2 days, with the highest and lowest variation at CCU and CL, respectively. However, the ALOS of emergency patients at CCU and PCCU is almost the same. The value of the coefficient of variation (CV) indicates that although the exponential distribution is appropriate for CCU, for other units, the LOS follows the distribution like the gamma or Erlang distribution. Except for the distribution at CL, each distribution has a moderately large trail.

**TABLE 2:** The descriptive statistics of length of stay (LOS) at each unit.

Unit	Mean	Median	Standard deviation (SD)	CV = SD/mean
NC	2.52	2	1.91	0.76
CL	2.19	2	1.01	0.46
CCU	2.93	2	2.97	1.01
PCCU	2.95	2	2.17	0.74

Abbreviations: CCU, Coronary Care Unit; CL, Cath Lab; CV, coefficient of variation; NC, Normal Care; PCCU, Progressive Coronary Care Unit.

Previous studies have explored the distribution of LOS in various hospital departments, indicating that gamma and Erlang distributions often provide a better fit than simple exponential models [31]. Furthermore, the importance of understanding LOS distributions to manage hospital bed capacities effectively has been highlighted [32], aligning with the findings this study.

**FIGURE 5:**
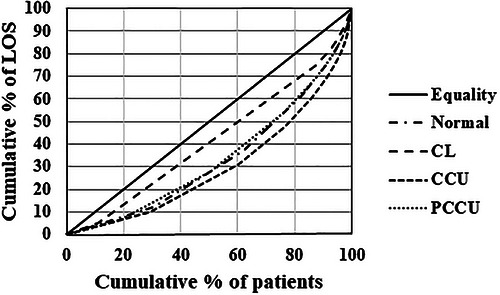
Lorenz curves LOS according to the number of patients staying in each unit. CCU, Coronary Care Unit; CL, Cath Lab; LOS, length of stay; PCCU, Progressive Coronary Care Unit.

By staying in healthcare facilities, patients utilize the resources that are available under the prevailing circumstances. To see the utilization ratio, the Gini coefficient of LOS among patients has been calculated for each unit. These values are 0.169, 0.324, 0.337, and 0.403 for CL, PCCU, NC, and CCU. Furthermore, the corresponding Lorenz curves are plotted in Figure 5. Both the coefficients and curves point out that in terms of LOS, the patients at CL are more homogeneous and those at CCU are most heterogeneous. The curves show that the top 20% longer stayers consume 33%, 40%, 42%, and 49% of resources at CL, PCCU, NC, and CCU, respectively. Longer stays for a subset of patients disproportionately strain resources, particularly in CCU and PCCU, which are critical for emergency and intensive care. These findings highlight the urgent need to optimize patient turnover rates and expand capacity.

### 3.4 The Performance of the Units

**FIGURE 6:**
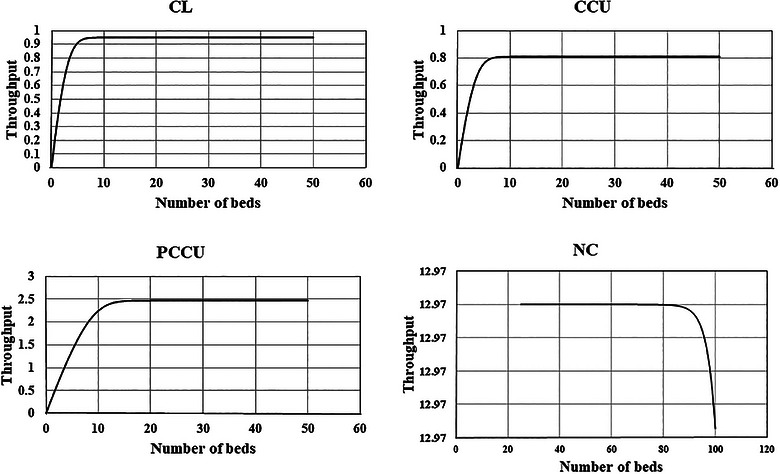
Variation of throughput for varying the numbers of beds at different units. CCU, Coronary Care Unit; CL, Cath Lab; NC, Normal Care; PCCU, Progressive Coronary Care Unit.

Using the selected queuing models (*M*/*M*/*c*/*c* and *M*/*M*/*c*/*K*) in Section 2.3 along with the extracted information in Sections 3.2 and 3.3 on arrival rates and LOS values, we have presented in this section the performance of each unit of the cardiology department. As the throughput of patients through a healthcare unit is an important issue to determine the real capacity of receiving and providing services to patients, it is considered the main performance measure. The throughputs for different numbers of beds are provided in Table 3 and visualized in Figure 6, respectively.

**TABLE 3:** Changes in throughput at each unit for various amount of beds.

**CL**	Total beds	3	4	5	6	7	8	9	10
	Throughput	0.74	0.85	0.92	0.94	0.95	0.95	0.95	0.95
**CCU**	Total beds	4	5	6	7	8	9	10	11
	Throughput	0.70	0.76	0.79	0.80	0.81	0.81	0.81	0.81
**PCCU**	Total beds	13	14	15	16	17	18	19	20
	Throughput	2.42	2.45	2.46	2.46	2.47	2.47	2.47	2.47
**NC**	Total beds (with 24 regular)	81	82	…				99	100
	Throughput	12.97	12.97	…				12.97	12.97

Abbreviations: CCU, Coronary Care Unit; CL, Cath Lab; NC, Normal Care; PCCU, Progressive Coronary Care Unit.

Comparing the actual arrival rate of 0.95 patients per day at CL with the throughput for the different number of beds, we can conclude that to receive and serve all arrival patients at this unit, on average, 7 beds are enough. As there are 10 beds available at this unit, the authority can shift 3 beds to other units where the scarcity of beds is prevalent. A similar comparison of the actual arrival rate of 0.81 patients per day with throughputs reveals that the CCU is utilizing the resources optimally. There is no way to reduce the bed in this unit. However, at PCCU, under the prevailing routine procedure of patients, there are not enough beds to meet the real demand. In addition to the existing 10 beds, at least 7 beds should be added and equipped to meet the existing flow of 2.47 patients per day. This targeted reallocation is essential for optimizing resource use to enhance access and equity in low‐resource settings.

Finally, no throughput at NC increases with the addition of extra beds (in addition to 24 regular beds). Although taking two digits after the decimal at the arrival rate does not show any change in throughput, it exhibits a slight deterioration in the effective arrival rate when 10 digits after the decimal are considered (Figure 6). This is rational because, under the peculiar condition of facilitating patients with extra beds, the NC unit is too overloaded. The concept of extra beds is helpful to avoid denial of admission for patients at the tertiary hospital. However, the unbounded exercise of such practice can collapse the healthcare system because the increase in the number of extra beds gives the patients the chance of staying a long time in the unit.

### 3.5 Sensitivity Analysis and Optimization

Optimization was performed by sensitivity analysis that varying the number of beds (servers) and evaluating system performance metrics such as throughput and blocking probability. The optimal configuration was identified as the minimum number of beds required to achieve near‐complete throughput (i.e., minimal patient rejection) under observed arrival rates.

**FIGURE 7:**
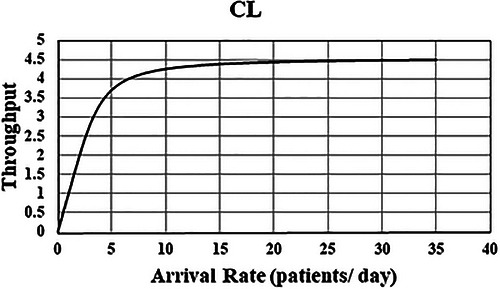
Variation of throughput for varying patient arrival rates at CL. CL, Cath Lab.

Figure 7 shows the expected performance or throughput against the patient arrival rate that is happening in the existing capacity of 10 beds at CL. It is evident that as the arrival rate increases, the throughput or performance also upturns to a certain level and finally becomes saturated. Up to around 1.5 patients per day can be received at the CL unit without any refusal of admission. Thus, without adding any beds, this unit can increase the rate of admission from the current practice of 0.95 patients per day. However, whatever the allowed rate of arrival and the percentage of refused admission (balking probability), the unit is not capable of receiving the patients at the rate of above 4.5 patients per day.

**FIGURE 8:**
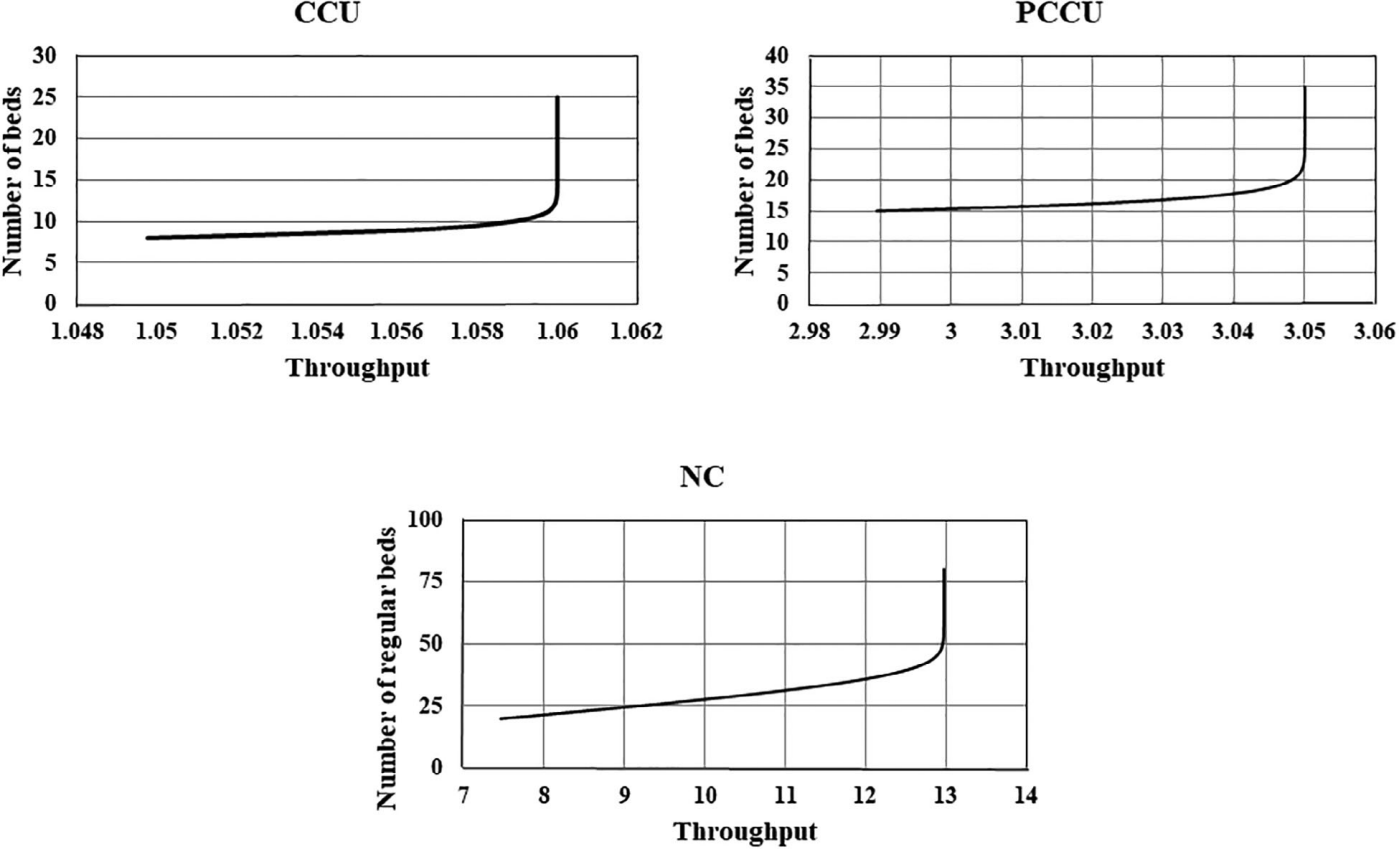
The number of the required regular beds for different throughput at (a) CCU, (b) PCCU, and (c) NC. CCU, Coronary Care Unit; NC, Normal Care; PCCU, Progressive Coronary Care Unit.

As noted in Section 3.2, the CCU unit should be equipped to provide treatment to a patient flow of 1.06 patients per day. In that case, the management needs to determine and manage the optimal number of beds in the unit. Using the ALOS and targeting uninterrupted throughput for the arrival rate of 1.06 patients per day, the number of required beds is plotted against the throughput in Figure 8a. It indicates that to fulfill the target, at least 15 beds are required.

After optimizing and improving the quality of CL and CCU, the emphasis should be given to improving the condition of the PCCU unit. With the optimal throughput of 1.5 patients per day at CL, the required throughput of 1.06 patients per day at CCU, and the absolute arrival of 0.49 patients per day, the PCCU unit should be replicated for the arrival of 3.05 patients per day. Like Figure 8a,b shows that at least 25 beds are required at PCCU to meet the target of optimization and improvement of the integrated system of healthcare. Such findings indicate that one additional unit of each CCU and PCCU is better to construct to provide the required treatment to the emergency patients in the secondary flow. Or else, the existing CCU and PCCU units could be merged and equipped to develop an extended CCU unit and a new PCCU unit with a capacity at least double the current one necessary to be constructed for delivering and promoting the intervention to prevent heart attacks in the catchment area of the hospital. That is, it is underscored the importance of expanding capacity in high‐demand units to prevent saturation and maintain service quality.

Finally, a sensitivity analysis for the NC unit suggests that to avoid overload of inpatient flow at this unit, the number of regular beds should be increased. Thus, Figure 8c, based on the *M*/*M*/*c*/*c* model, shows that instead of 24 regular beds and 49 extra beds, around 50 regular beds are enough to meet the existing pressure of 12.97 patients per day at NC. Again, a new additional NC unit with the same capacity as the existing one is essential to the healthcare chain of the cardiology department. Overall, the study highlights the critical need for capacity expansion in high‐demand units to prevent overcrowding and maintain high‐quality healthcare services.

Although increasing bed capacity can enhance service levels and alleviate congestion, it also incurs substantial operational and capital costs, including staffing, equipment, and infrastructure expansion. Therefore, capacity‐related decisions must balance improvements in service delivery with financial constraints. The findings support a targeted strategy that prioritizes high‐impact units such as CCU and PCCU, while simultaneously improving system efficiency through the reallocation of underutilized resources, such as beds from CL, to limit additional costs.

Consistent with prior research, the results indicate that hospital congestion largely arises from imbalances between arrival rates and service capacity. However, this study extends existing work by emphasizing the interdependence among multiple cardiac units, particularly the cascading effect whereby congestion in CCU contributes to overload in PCCU. From a managerial perspective, the analysis offers clear operational guidance: Reallocating underutilized CL beds can improve overall system balance, targeted expansion of CCU and PCCU capacity is necessary to reduce emergency overflow, and the unregulated use of extra beds in NC should be replaced with structured capacity planning. Collectively, these insights provide a practical, evidence‐based framework to support resource allocation decisions in resource‐constrained healthcare settings.

On the basis of the findings, this study provides three key contributions. First, it demonstrates how queuing theory can be adapted to capture informal practices such as overflow bed usage in low‐resource hospitals. Second, it identifies critical bottlenecks in emergency cardiac care, particularly the mismatch between CCU demand and capacity. Third, it offers actionable and context‐sensitive strategies, including redistribution of underutilized resources and targeted expansion of high‐demand units, thereby moving beyond generic recommendations toward implementable solutions.

## 4 Conclusions

This study demonstrates the critical need for optimizing inpatient flow and resource allocation in the cardiology department of Sylhet MAG Osmani Medical College Hospital, a key healthcare facility in a low‐resource setting. By applying queuing theory models (*M*/*M*/*c*/*c* and *M*/*M*/*c*/*K*) to real‐world data from 6277 patients treated in 2023, our findings underscore systemic inefficiencies that hinder equitable access to cardiac care, especially for low‐income populations who rely heavily on public healthcare. In addition, the models have been found to be robust tools for assessing the performance of the integrated healthcare system.

The analysis reveals that although the NC unit accommodates the majority of patients, the pervasive use of extra beds compromises care quality and creates operational challenges. In particular, the CCU and PCCU experience severe bed shortages, with occupancy exceeding 100%, highlighting an urgent need for capacity expansion. The study identifies a requirement of 15 beds in the CCU and 25 beds in the PCCU to address unmet demand and improve emergency care during critical periods, such as the “Golden Hour,” where timely intervention is crucial for survival.

Through sensitivity analysis, the study further establishes the need for dynamic resource reallocation. For example, underutilized beds from the CL can be shifted to other units to mitigate shortages. Additionally, our analysis of arrival patterns and LOS reveals significant heterogeneity in resource utilization, with longer stays disproportionately straining CCU and PCCU resources. Addressing this requires targeted interventions to optimize patient turnover rates and streamline care delivery.

Although increasing bed capacity can improve system performance, such expansion must be considered alongside practical constraints such as staffing, infrastructure, and financial limitations. Therefore, this study emphasizes a combination of strategies, including resource reallocation (e.g., reallocating underutilized beds from CL), improved patient routing, and targeted capacity expansion in critical units, rather than indiscriminate increases in beds. The study findings align with broader global health objectives of achieving universal health coverage and reducing healthcare disparities.

## 5 Limitations and Future Research

This study, focused on optimizing inpatient flow and resource allocation in the cardiology department of a tertiary hospital in a low‐resource setting, has several limitations. First, it primarily relies on data from a single hospital, which may not fully represent the diverse challenges faced by similar facilities in other regions of Bangladesh or developing countries. Second, although queuing theory models were effective in analyzing patient flows, additional modeling approaches, such as agent‐based simulations, could provide deeper insights into dynamic interactions within the healthcare system. Future research should extend this approach to include multi‐hospital systems, examine the impact of socioeconomic disparities, and evaluate the integration of digital health technologies for real‐time resource management.

## Author Contributions

**Md. Borhan Uddin**: conceptualization, data curation, formal analysis, visualization, roles/writing – original draft. **Khalidur Rahman**: conceptualization, formal analysis, methodology, project administration, supervision, validation, visualization, roles/writing – original draft, writing – review and editing. **Md. Asaduzzaman**: conceptualization, roles/writing – original draft, writing – review and editing. **Sabbir Tahmidur Rahman**: conceptualization, roles/writing – original draft, writing – review and editing. **Md. Sabbir Hossain**: data collection, data validation, literature review, writing – review and editing. **Md. Shihab Mostafa**: data collection, literature review, writing – review and editing.

## Funding

The authors have nothing to report.

## Ethics Statement

This study optimizes inpatient flow in a cardiology department using queuing theory, relying solely on retrospective, anonymized hospital records. As it involves no direct patient interaction or experimental interventions, there are no risks to patient privacy or well‐being. Consequently, ethics committee or Institutional Review Board approval is unnecessary, because the research adheres to ethical standards for non‐interventional studies using existing, anonymized data.

## Consent

The authors have nothing to report.

## Conflicts of Interest

The authors declare no conflicts of interest.

## Data Availability

The data that support the findings of this study are available on request from the corresponding author. The data are not publicly available due to privacy or ethical restrictions.
